# 

**Published:** 1934

**Authors:** 


### The Medical Library of the University of Bristol,

WITH WHICH IS INCORPORATED

**The Library of the Bristol Medico-Chirurgical Society.**

---

*The following donations have been received since the publication of the list in March,* 1934.

June, 1934.

| | |
|---|---|
| J. Michell Clarke Fund (1) .. .. .. | 4 volumes. |
| Genatosan Ltd. (2) .. .. .. .. | 1 volume. |
| General Medical Council (3) .. .. .. | 3 volumes. |
| Otago University (4) .. .. .. .. | 1 volume. |
| Pennsylvania University (5) .. .. .. | 1 ,, |
| Mrs. Sturmer (6) .. .. .. .. | 4 volumes. |
| Dr. P. Watson-Williams (7) .. .. .. | 1 volume. |

Unbound periodicals have also been received from Professor E. W. Hey Groves and Dr. C. Bruce Perry.

### THE ONE HUNDRED AND FORTY-SEVENTH LIST OF BOOKS.

The figures in round brackets refer to the figures after the names of the donors. The books to which no such figures are attached have either been bought from the Library Fund or received through the *Journal.*

**Auty, C. H.** .. .. .. *Modern Inhalation Therapy* .. .. .. 1933

**Aykroyd, W. R.** .. .. *Vitamins and other Dietary Essentials* .. 1933

**Bailey, P.** .. .. .. *Intracranial Tumours* .. .. .. .. (1) 1933

**Bayly, H. W.** .. .. *Venereal Disease* .. .. .. .. 5th Ed. 1934

**Beaumont, G. E., and Dodds, E. C.** *Recent Advances in Medicine* 7th Ed. (1) 1934

**Bett, W. R., ed.** .. .. *Short History of Some Common Diseases by Divers Authors* .. .. .. .. .. 1934

**Blacker, C. P. ed.** .. *Chances of Morbid Inheritance* .. .. .. 1934

**Botey, R.** .. .. .. *Les Tumeurs Bénignes du Larynx* .. (7) [1932]

**Boyd, W.** .. .. .. *Textbook of Pathology* .. .. .. .. .. 1932

**Bryan, C. P.** .. .. .. *Papyrus Ebers* .. .. .. .. .. .. .. [1930]

*Cambridge Natural History*, Vols. 2 and 5 .. .. .. .. (6) 1895–1896

**Cole, A. C.** .. .. .. *Studies in Microscopical Science*, Vol. 1 (6) 1883

**Cox, G. M.** .. .. .. *Clinical Contraception* .. .. .. .. .. 1933

**Cushny, A. R.** .. .. *Textbook of Pharmacology and Therapeutics* 10th Ed. (1) 1934

**Dean, E.** .. .. .. *Spadacrene Anglica, or the English Spaw* 1736

**Gask, G. E., and Ross, J. P.** *Surgery of the Sympathetic Nervous System* .. .. .. .. .. .. .. (1) 1934

**Gibson, A. G.** .. .. *Physician's Art* .. .. .. .. .. .. .. 1933

**Giles, G. M.** .. .. .. *Handbook of the Gnats or Mosquitoes* (6) 1900

**Gunn, J. A.** .. .. .. *Introduction to Pharmacology and Therapeutics* .. .. .. .. 3rd Ed. 1932

**Harrison, G. A.** .. .. *Chemical Methods in Clinical Medicine* .. 1930

**Hentschel, C. C., and Cook, W. R. I.** *Biology for Medical Students* .. 1932

**Hutton, I. E.** .. .. *Hygiene of Marriage* .. .. .. 4th Ed. 1933

**McAusland, S.** .. .. *Cure of Hæmorrhoids* .. .. .. .. .. 1933

**Pachon, V., and Fabre, R.** *Clinical Investigation of Cardiovascular Function.* Transl. by J. F. H. Dally 1934

**Pearl, R.** .. .. .. *Constitution and Health* .. .. .. .. 1933

**Ray, M. B.** .. .. .. *Rheumatism in General Practice* .. .. .. 1934

**Statham, R. S. S.** .. *Outline of Practical Obstetrics for Nurses* 1933

*This Panel Business*, by A. G. P. .. .. .. .. .. .. .. .. .. 1933

**Thomson, J., and Findlay, L.** *Clinical Study and Treatment of Sick Children* .. .. .. .. .. 5th Ed. 1933

**Webb, P. M.** .. .. *Diets* .. .. .. .. .. .. .. .. .. (2) [1934]

TRANSACTIONS, REPORTS, JOURNALS, ETC.

Bristol Medico-Chirurgical Journal. Combined Index for Vols 1 to 50, 1883–1933 .. .. .. .. .. .. .. .. .. .. .. .. .. .. [1934]

Dentists' Register .. .. .. .. .. .. .. .. .. .. .. .. .. .. (3) 1934

Medical Annual .. .. .. .. .. .. .. .. .. .. .. .. .. .. .. (3) 1934

Medical and Dental Student's Register .. .. .. .. .. .. .. (3) 1933

Medical Register .. .. .. .. .. .. .. .. .. .. .. .. .. .. (3) 1934

Otago University. Proceedings of the Medical School, No. 11 .. (4) 1934

Pennsylvania University. Henry Phipps Institute, 24th Report, 1932–33 .. .. .. .. .. .. .. .. .. .. .. .. .. .. .. (5) 1933

Quarterly Cumulative Index Medicus, Vol. 14 .. .. .. .. .. .. 1933

Surgical Clinics of North America, Vol. 14, Nos. 1 and 2 .. .. .. 1934

### Publications Received

From EDWARD ARNOLD :
*Manipulative Treatment for the Medical Practitioner.* By T. MARLIN, M.D. Price 10s. 6d.

From BAILLIERE, TINDALL & COX :
*Aids to Obstetrics,* 10th Ed. By L. WILLIAMS, M.D., M.S. Price 3s. 6d.
*Aids to Operative Surgery.* Ed. 2. By C. P. G. WAKELEY, D.Sc. Price 3s. 6d.

From WILLIAM HEINEMANN LTD. :
*Handbook of Filterable Viruses.* By R. W. FAIRBROTHER, M.D. Price 7s. 6d.

From H. K. LEWIS & CO. LTD. :
*The Influence of Heredity on Disease.* By L. S. PENROSE, M.A., M.D. Price 5s.
*Surgical Anatomy and Physiology.* By N. C. LAKE, M.D., M.S., D.Sc. and C. J. MARSHALL, M.D., M.S. Price 30s.

From E. & S. LIVINGSTONE :
*Vital Cardiology.* By B. WILLIAMSON, M.D. Price 15s.
*Advice to the Expectant Mother.* Ed. 3. By F. T. BROWNE, M.D., D.Sc. Price 6d.
*An Introduction to Practical Bacteriology.* By T. J. MACKIE, M.D., and J. E. MCCARTNEY, M.D., D.Sc. Price 12s. 6d.

From MEDICAL PUBLICATIONS LTD. :
*Abscess of the Brain.* By E. M. ATKINSON, M.B., B.S. Price 21s.

From OXFORD UNIVERSITY PRESS :
*Applied Physiology.* 5th Ed. By SAMSON WRIGHT, M.D. Price 18s.
*The Anœmias.* By J. M. VAUGHAN, D.M. Price 12s. 6d.
*Nephritis and Allied Diseases.* By ROBERT PLATT, M.D. Price 7s. 6d.

From JOHN WRIGHT & SONS LTD. :
*Essays on Chronic and Familial Syphilis.* By GRIFFITH EVANS, M.A., D.M. Price 7s. 6d.

### Local Medical Notes

**Long Fox Memorial Lecture.**—The twenty-third Long Fox Memorial Lecture will be delivered at the University on Tuesday, 3rd July, 1934, at 5 p.m., by Dr. F. J. Poynton, F.R.C.P. Subject: "Some Aspects of Heart Disease in Children."

**Colston Medical Research Fellowships.**—The Council of the Colston Research Society, Bristol, offers one or more Fellowships for 1934-35 of not less than £100 per annum for research work primarily in Clinical subjects, though other medical subjects are not excluded. The work must be carried out in the University of Bristol or in any of the Clinical Institutions associated with the University.

Awards will be made in June or July, 1934. Applications should be forwarded forthwith to the Hon. Secretary of the Colston Research Society.

EXAMINATION RESULTS.

**University of Bristol.**—Students of the University have recently passed the following examinations:—

M.D.—*Pass with Distinction:* H. Rogers. *Pass:* F. H. Bodman, J. J. J. Giraldi, N. L. Price.

Ch.M.—*Pass with Distinction:* G. F. Langley.

M.B., Ch.B.—*Final Examination* (*Part II.*). *Pass* (*First Class Honours*): A. C. Molden (with Distinction in Surgery, Obstetrics and Public Health. *Pass* (*Second Class Honours*): A. G. W. Branch (with Distinction in Obstetrics), Rosalind M. S. Derham (with Distinction in Public Health), C. H. G. Price. *Pass:* Grace J. V. Ball, Dorothy E. Barber, Violet Fry, N. Greenberg, G. L. L. Gurney, T. R. V. Gurney, Gwladys R. Llewelyn, R. A. Mathews (with Distinction in Surgery and Public Health).

M.B., Ch.B.—*Final Examination* (*Part I.*). *Pass:* M. A. Nicholson, B. Ridgway.

M.B., Ch.B. (*Part II.*).—*Second Examination. Pass :* Daphne V. Dennis, R. L. J. Derham, P. K. Jenkins, Margaret E. Morgan.

M.B., Ch.B. (*Part I.*).—*Second Examination. Pass :* K. A. Hall, J. S. Richardson.

B.D.S.—*Third Examination. Pass :* C. A. Blanden (with Distinction in Dental Mechanics), A. N. Brimelow, C. C. B. Plumley, P. R. Quartley.

B.D.S.—*Second Examination. Pass :* P. H. S. Paine, F. P. Ewens.

L.D.S.—*Second Professional Examination. Pass :* R. E. Buchan, J. G. Coates, T. John, C. L. Read, A. J. Staple.

L.D.S.—*First Professional Examination. Pass :* M. G. Davies, H. W. Williams, R. J. Lee, H. B. Harding, H. D. Williams.

L.D.S.—*Preliminary Science Examination. Pass :* D. C. Bodenham, P. Cairns, C. F. Howell, A. J. H. Orchard, Peggy M. Rooke, D. M. Sanders.

**Conjoint Board.**—M.R.C.S., L.R.C.P.—*Pass : In Chemistry and Physics :* A. R. Bird. *In Anatomy :* W. White, Mabel L. Glenny. *In Anatomy and Physiology :* F. D. Mowat. *In Medicine :* C. H. G. Price,* Rosalind M. S. Derham,* J. N. Heales.* *In Medicine and Surgery :* A. G. W. Branch,* R. H. Moodie.* *In Surgery :* Dorothy J. Thompson, R. A. Mathews, Violet Fry.* *In Midwifery :* L. E. Sealy, E. J. Perks, S. Roberts. *In Pathology :* Irene G. Hamilton.

L.D.S., R.C.S.—*First Professional Examination. Pass : Part I :* J. G. Jones *Part II. :* E. F. Sumner, J. R. P. Thomas. *Part III. (b) :* D. J. Dymond.

* Qualified.

